# Impact of Oxycodone HCl Extended-release Formulary Restrictions on Extended-release Opioid Market Share, Healthcare Resource Utilization and Costs in Commercial and Medicare Plans

**DOI:** 10.36469/9800

**Published:** 2017-07-11

**Authors:** Rolin L. Wade, Chi-Chang Chen, Ajita P. De, Jaren C. Howard

**Affiliations:** 1 QuintilesIMS, Plymouth Meeting, PA; 2 Purdue Pharma L.P., Stamford, CT

**Keywords:** health plans, healthcare costs, utilization management, formulary restrictions, oxycodone hcl extended release, opioids

## Abstract

**Background:** Previous research demonstrated that utilization management (UM) such as prior authorization (PA) or non-formulary (NF) restrictions may reduce pharmacy costs when designed and applied appropriately to certain drug classes. However, such access barriers may also have unintended consequences. Few studies systemically analyzed the impact of major UM strategies to extended-release (ER) opioids on different types of health plans.

**Objective:** This study evaluated, from payer perspective, the impact of formulary restrictions (PA, NF, or step therapy [ST]) for branded oxycodone HCl extended release (OER) on market share, and healthcare resource utilization/costs in ER opioids patients for multiple types of health plans in the United States.

**Methods:** This retrospective, longitudinal case-control study analyzed prescription and outpatient medical claims data (2012 to 2015) for adult ER opioid patients from US plans (commercial,/Medicare, national/regional) that instituted OER PA, NF, or ST. Patients from each restricted plan (cases) were matched to patients in an unrestricted plan (controls) on key patient characteristics. ER opioid market share and healthcare resource utilization/costs for both cases and controls were evaluated for the 6-month period before and after the formulary restriction dates. A difference-in-differences (DiD) approach was utilized to evaluate change in the total per patient per month (PPPM) healthcare utilization and costs.

**Results:** The study comprised 1622 (national commercial PA), 2020 (regional commercial PA), 34 703 (national commercial ST), and 4372 (national Medicare NF) cases and equivalent number of controls. OER market share decreased after the formulary restrictions, with the national Medicare NF plan showing the greatest decrease (9.2%). DiD analyses indicated that PPPM office visit change in the PA and NF plans were non-significant (decreased by 0.1 and 0.2, P>0.05), but significant in the ST plan (increased by 0.1, P=0.0001). For most plans, no significant total monthly cost change was observed; PPPM costs decreased by $48.74 and $59.87 in ST and regional PA plans and increased by $37.90 in national NF plans (all P>0.05).

**Conclusions:** This study observed that despite reducing the market share of OER, OER formulary restrictions had negligible impact on overall ER opioid utilization, and did not result in substantial pharmacy/medical cost savings.

## INTRODUCTION

Utilization management through formulary restrictions (such as prior authorization [PA], step therapy [ST], and non-formulary [NF] edits) is designed to reduce pharmacy costs and promote safe and appropriate drug utilization.[1] Payer formulary restrictions vary in requirements before a drug is authorized for payment. For example, a PA may require providers to demonstrate medical necessity by documenting a diagnosis, lab value, prerequisite therapy, or a combination of these before the drug is approved for the patient. ST typically requires patients to try a prerequisite preferred agent before approval of a "step" up to a non- preferred drug. With NF restrictions, certain drugs are excluded from the health plan formulary and require the patient to go through an exception process to obtain coverage.

Some studies have shown that formulary restrictions affect medication utilization patterns and reduce pharmacy costs.[2,3,4] These restrictions tend to be most successful for drug classes that possess similar clinical efficacy and relatively homogenous patient therapeutic responses, such as HMG-CoA reductase inhibitors (statins) or estrogens.[5,6] However, other studies have documented that various types of restrictions across diverse therapeutic categories resulted in negative impact on patient's health status, or did not offer substantial cost reductions when total, not just pharmacy, cost is considered.[7,8,9] Outcomes and costs of formulary restrictions are known to vary by payer segment, plan size, region, drug category, disease state, as well as by provider and member response to formulary changes.

As of 2011, more than 115 million adult Americans suffer from chronic pain conditions; and is expected to increase with an aging population and longer lifespans.[10,11] The economic burden of chronic pain management has been estimated at $560 to $635 billion annually, or approximately $2000 for every US resident. Chronic pain patients are a vulnerable and challenging population to treat.[10] These individuals often present with physical and psychological comorbidities and require a multi-modal, interdisciplinary approach, including, for some chronic pain patients, appropriate use of opioids. Branded oxycodone HCl extended-release (OER) is an extended-release opioid (ER opioid) currently approved by the US Food and Drug Administration (FDA) for the management of pain severe enough to require around-the-clock, long-term opioid treatment, and for which alternative treatment options are inadequate.[12]

Despite the institution of formulary restrictions on opioids used for non-malignant, chronic pain management, the effects on utilization and total costs are not clear. In 2014, Ben-Joseph et al evaluated the effects of formulary restrictions of OER for patients with chronic non-malignant pain in both commercial and Medicare plans and found mixed results in resource utilization and costs which suggested that restrictions may lead to unintended consequences such as increased costs.[9] In general, evidence to date is very limited regarding the impact that ER opioid formulary restrictions may have on utilization and or economic outcomes. This study sought to evaluate, from the health plan perspective, the impact of formulary restrictions (PA, ST, NF) for OER on market share, resource utilization and costs when applied across multiple commercial (regional and national) and Medicare Part D health plans.

## METHODS

### Study Design

This retrospective, longitudinal case-control study used a difference-in-differences (DiD) design to evaluate the impact of OER formulary restrictions on ER opioid market share and healthcare utilization/costs among commercial and Medicare health plans. Separate analyses of longitudinal pharmacy and medical claims data were conducted for the following case plans with specific formulary restrictions: 1) a national commercial plan with a PA (National Commercial PA); 2) a regional commercial plan with a PA (Regional Commercial PA) but allowed grandfathering of OER users; 3) a national commercial plan with ST (National Commercial ST); and 4) a national Medicare plan with the drug not on formulary (National Medicare NF). Patients in each case plan were matched to patients in a control plan without OER formulary restriction in the same time frame as the case plans.

### Data Source

Study data were obtained from the QuintilesIMS patient centric Pharmacy Claims Database and Medical Claims Database. Data from the Pharmacy Claims Database has 86% coverage of the retail channel, 55% of standard mail service and 40-70% of specialty pharmacy volume. This database includes all payment types including cash, Medicaid, Medicare, and all third-party transactions. While this data source is useful due to its broad coverage of prescription claims, and is payer agnostic, the lack of an eligibility file requires observation of claims to establish a longitudinal record for the patient. We apply an observation of claims before and after the study period for each patient to establish confidence in longitudinal records.

The QuintilesIMS patient centric Medical Claims Database is derived from professional fee claims using the CMS-1500 billing form. It provides patient-level diagnoses, procedures for visits to US office-based physicians, ambulatory, and general health care sites and un-adjudicated charge data. This amounts to >1 billion claims per year, representing over 860,000 providers per month.

### Sample Selection

Eligible cases were enrollees with ER opioid use (≥1 ER opioid prescription in the pre-restriction period). The study duration for each plan evaluated was 1 year, and patients were required to be observed and have complete pharmacy/medical data during the entire study period. The year was determined by the date that a plan's restriction began, with a 6-month pre-restriction and a 6-month post-restriction period on either side of the restriction date. Study periods were as follows: National Commercial PA (7/1/2013-6/30/2014; restriction date 1/1/2014); Regional Commercial PA (1/1/2012-12/31/2012; restriction date 7/1/2012); National Commercial ST (1/1/2015-12/31-2015; restriction date 7/1/2015); and, National Medicare NF (7/1/2012-6/30/2013; restriction date 1/1/2013). Because there were no plan enrollment files available within the QuintilesIMS patient centric databases, continuous enrollment was proxied by requiring at least one claim for any prescription in the 3-month periods before and after both ends of the 1-year study period.

Eligible patients were required to be ≥18 years of age upon the first ER opioid claim. Control patients were chosen as enrollees in a plan similar to the restrictive plan based on plan size and segment (national/regional, Commercial/Medicare) without OER formulary restrictions and with ER opioid use in the same pre-restriction period. Cases and controls were matched on key clinical and demographic characteristics (patient age within a 5-year interval; sex [male/female]; geographic region [Northeast, West, Midwest, South]; Charlson Comorbidity Index score [CCI; 0, 1-2, 3-5, ≥6]; cancer vs non-cancer diagnosis [pre-restriction period]; new vs continued LAO users [pre-restriction period]), and were followed for 6 months in the post-restriction period.

### Measures and Analysis

ER opioid market share was calculated by dividing the total number of prescriptions observed for the ER opioid group in question by the total number of prescriptions observed for all ER opioids in the pre-restriction and post-restriction periods. OER vs other ER opioid share change was calculated during the follow-up period (comparing 6 months pre-restriction versus 6 months post-restriction).

Healthcare resource utilization and costs were captured for both the pre- and post-restriction periods for both cases and controls. Per patient per month (PPPM) utilization was measured using the count of pharmacy prescriptions dispensed and the count of outpatient claims (office and other visits) during the 6-month pre and post periods. Additionally, costs were captured as pharmacy charges for all prescriptions (ER opioid, short-action opioid [SAO], and non-opioid medications) and charges for outpatient claims (office and other visits). The difference in mean pre-/post- changes were compared between cases and controls to evaluate the adjusted net impact of OER access restriction on resource utilization and costs.

Bootstrapping t-test and generalized linear models with gamma distribution and a log-link were utilized to test the pre- and post- differences (within cases/controls, and between cases/controls) in resource utilization and costs, respectively. All costs were adjusted to 2014 dollars using the Medical Care Consumer Price Index for All Urban Consumers.

## RESULTS

The final sample comprised 1622 (national commercial PA), 2020 (regional commercial PA), 34 703 (national commercial ST), and 4372 (national Medicare NF) chronic ER opioid users, and equivalent number of controls from no restriction commercial and Medicare control plans with similar regional/national and health plan type (Table 1).

In the national commercial PA plan, OER market share decreased by 7.0% and the total PPPM decrease from pre to post index was $234.83 (after adjusting for changes in the control plan). In the Regional commercial PA plan, OER market share decreased by 0.3% and the total PPPM decrease from pre to post index was $59.87. In the national commercial ST plan, OER market share decreased by 0.3% and the total PPPM increase from pre to post-index was $48.74. In the national Medicare NF plan, OER market share decreased by 9.2% and the total PPPM increase from pre to post index was $37.90. More detailed results are discussed below.

Post case-control matching, the National Commercial PA cohort (mean age 49.1; 56.0% female), 4.0% were cancer patients and the mean CCI score was 0.61; in the Regional Commercial PA cohort (mean age 56.6; 54.4% female), 1.1% were cancer patients and the mean CCI score was 0.1; in the National Commercial ST cohort (mean age 58.4; 61.7% female), 1.8% were cancer patients and the mean CCI score was 0.2; in the National Medicare NF cohort (mean age 61.3; 63.4% female), 1.6% were cancer patients and the mean CCI score was 0.2. A complete breakdown of pre- and post-matching demographic and clinical characteristics can be found in Table 2.

**Table 1.** Patient Attrition Chart

| | | National Commercial (Study period 7/1/2013-6/30/2014) | | Regional Commercial (Study period 1/1/2012-12/31/2012) | | National Commercial (Study period 1/1/2015-12/31/2015) | | National Medicare (Study period 7/1/2012-6/30/2013) | |
|---|---|---|---|---|---|---|---|---|---|
| **STEP** | **Criteria** | **CASE (PA)** | **Control** | **CASE (PA)** | **Control** | **CASE (ST)** | **Control** | **CASE (NF)** | **Control** |
| **1** | Patients in QuintilesIMS pharmacy claims database enrolled in the Plan of interest for entire study period | 456 341 | 1 099 488 | 588 631 | 2 465 534 | 5 824 693 | 3 527 340 | 680 953 | 542 413 |
| **2** | Patients 18 years of age or older at the beginning of study period, AND with valid gender information | 390 665 | 936 384 | 509 268 | 2 214 236 | 5 784 915 | 3 202 884 | 674 498 | 536 292 |
| **3** | Patients with ≥1 ER opioid claims from the Plan of interest in the pre-restriction 6-month period in QuintilesIMS pharmacy claims database | 3372 | 10 277 | 4497 | 55 069 | 172 163 | 70 328 | 19 403 | 14 661 |
| **4** | Patients pharmacy activities were continuously captured by QuintilesIMS throughout the study data period | 2831 | 8771 | 4313 | 50 675 | 166 312 | 67 645 | 18 550 | 13 873 |
| **5** | Patients from Step 4 matched to QuintilesIMS medical claims database during study period | 2690 | 8039 | 3417 | 36 857 | 139 454 | 55 178 | 16 256 | 11 357 |
| **6** | Patient medical claims activities were continuously captured by QuintilesIMS during study period | 1918 | 5032 | 2046 | 19 912 | 92 832 | 35 075 | 10 474 | 7046 |
| **7** | Eligible patients after direct matching between case and controls by key clinical/demographic characteristics | 1622 | 1622 | 2020 | 2020 | 34 703 | 34 703 | 4372 | 4372 |

ER opioid: extended-release opioid; NF: non-formulary; PA: prior authorization; ST: step therapy

**Table 2.** Patient Baseline Characteristics

| | National Commercial PA | | National Commercial Control | | | Regional Commercial PA | | Regional Commercial Control | | | National Commercial ST | | National Commercial Control | | | National Medicare NF | | National Medicare Control | | |
|---|---|---|---|---|---|---|---|---|---|---|---|---|---|---|---|---|---|---|---|---|
| **Baseline Characteristics** | **n** | **%** | **n** | **%** | **P-value** | **n** | **%** | **n** | **%** | **P-value** | **n** | **%** | **n** | **%** | **P-value** | **n** | **%** | **n** | **%** | **P-value** |
| Sample Size (n) | 1622 | 100 | 1622 | 100 | NA | 2020 | 100 | 2020 | 100 | NA | 34 703 | 100 | 34 703 | 100 | NA | 4372 | 100 | 4372 | 100 | NA |
| **Demographic Characteristics** | | | | | | | | | | | | | | | | | | | | |
| **Age** | | | | | | | | | | | | | | | | | | | | |
| Mean | 49.1 | | 49.1 | | 0.9678 | 56.56 | | 56.57 | | 0.9784 | 58.36 | | 58.28 | | 0.3857 | 61.31 | | 61.26 | | 0.8528 |
| Median | 50.0 | | 50.0 | | | 56.00 | | 56.00 | | | 58.00 | | 58.00 | | | 61.00 | | 61.50 | | |
| SD | 9.9 | | 9.9 | | | 13.35 | | 13.42 | | | 12.57 | | 12.58 | | | 13.25 | | 13.26 | | |
| Sex | 0.9999 | | | 0.9999 | | | 0.9999 | | 0.9999 | | | | | | | | | | | |
| Female | 915 | 56 | 915 | 56 | | 1099 | 54.41 | 1099 | 54.41 | | 21 396 | 61.65 | 21 396 | 61.65 | | 2773 | 63.43 | 2773 | 63.43 | |
| Male | 707 | 44 | 707 | 44 | | 921 | 45.59 | 921 | 45.59 | | 13 307 | 38.35 | 13 307 | 38.35 | | 1599 | 36.57 | 1599 | 36.57 | |
| **Region** | | | | | 0.9999 | | | | | 0.9999 | | | | | 0.9999 | | | | | 0.9999 |
| Midwest | 441 | 27 | 441 | 27 | | 40 | 1.98 | 40 | 1.98 | | 5859 | 16.88 | 5859 | 16.88 | | 319 | 7.30 | 319 | 7.30 | |
| Northeast | 4 | 0 | 4 | 0 | | 1761 | 87.18 | 1761 | 87.18 | | 4984 | 14.36 | 4984 | 14.36 | | 332 | 7.59 | 332 | 7.59 | |
| South | 1015 | 63 | 1015 | 63 | | 170 | 8.42 | 170 | 8.42 | | 18 993 | 54.73 | 18 993 | 54.73 | | 2125 | 48.60 | 2125 | 48.60 | |
| West | 162 | 10 | 162 | 10 | | 49 | 2.43 | 49 | 2.43 | | 4867 | 14.02 | 4867 | 14.02 | | 1596 | 36.51 | 1596 | 36.51 | |
| **Clinical Characteristics** | | | | | | | | | | | | | | | | | | | | |
| **Diagnosis** | | | | | | | | | | | | | | | | | | | | |
| Cancer | 67 | 4% | 67 | 4% | 0.9999 | 23 | | 23 | | 0.9999 | 626 | 1.80 | 626 | | 0.9999 | 68 | 1.56 | 68 | 1.56 | 0.9999 |
| **Charlson Comorbidity Index score** | | | | | | | | | | | | | | | | | | | | |
| Mean | 0.61 | | 0.58 | | 0.8975 | 0.10 | | 0.10 | | 0.925 | 0.17 | 0.17 | | | 0.15 | | 0.15 | | | |
| Median | 0.00 | | 0.00 | | | 0 | | 0 | | | 0 | 0 | | | 0 | | 0 | | | |
| SD | 1.30 | | 1.19 | | | 0.67 | | 0.67 | | | 0.84 | 0.84 | | | 0.78 | | 0.78 | | | |

NF: not on formulary; PA: prior authorization; ST: step therapy

## ER Opioid Market Share Changes

Market share for OER decreased after the formulary restrictions for all study health plans, although the extent of decrease varies from less than 1% in Regional Commercial PA and National ST plans, to 7.0% and 9.2% in National Commercial PA and National Medicare NF plans, respectively. Detailed market share changes for each study plan are described below.

In the National Commercial PA plan, the OER market share dropped by 7.0% (from 31.2% to 24.2%) during the 6-month post-restriction period; during the same period, the control plan had a 1.4% decrease in OER market share (Table 3). In this National Commercial PA plan which restricted OER, market share appeared to shift mostly toward morphine ER generics (3.8% increase) and Fentanyl generics (2.3% increase); other ER opioid share changes were negligible (<1%).

**Table 3.** Change In Extended-release Opioid Market Share Across Health Plans Post OER Formulary Restrictions

| ER opioid prescription | National Commercial PA % Change in Market Share* | | Regional Commercial PA % Change in Market Share* | | National Commercial ST % Change in Market Share* | | National Medicare NF % Change in Market Share* | |
|---|---|---|---|---|---|---|---|---|
| | Cases | Controls | Cases | Controls | Cases | Controls | Cases | Controls |
| OxyContin (oxycodone HCl ER) | -6.98% | -1.37% | -0.28% | 0.17% | -0.29% | -0.62% | -9.24% | -0.02% |
| Avinza (morphine sulfate) | -0.18% | -0.34% | 0.12% | 0.00% | -0.12% | -0.08% | 0.33% | -0.03% |
| Butrans (buprenorphine) | 0.16% | -0.64% | -0.13% | -0.22% | -0.45% | -0.54% | -0.12% | -0.09% |
| Duragesic (fentanyl) | 0.05% | 0.09% | 0.01% | -0.02% | 0.01% | 0.00% | 0.03% | 0.10% |
| Exalgo (hydromorphone HCl) | -0.27% | 0.24% | 0.16% | 0.07% | -0.07% | -0.03% | 0.28% | 0.18% |
| MS Contin (morphine sulfate) | 0.00% | 0.06% | -0.01% | 0.00% | 0.00% | 0.00% | 0.00% | 0.02% |
| Nucynta ER (tapentadol HCl) | -0.04% | 0.07% | 0.09% | 0.03% | -0.07% | 0.04% | 0.07% | -0.08% |
| Opana ER (oxymorphone HCl) | 0.50% | 0.04% | 0.01% | -0.59% | 0.06% | -0.15% | 2.11% | -0.17% |
| Kadian (morphine sulfate) | -0.12% | -0.20% | -0.04% | -0.21% | 0.00% | 0.00% | -0.32% | -0.07% |
| Embeda (morphine-naltrexone) | 0.00% | 0.00% | 0.00% | 0.00% | 0.05% | 0.06% | 0.00% | 0.00% |
| Fentanyl Generics | 2.31% | 0.02% | -0.36% | -0.15% | -0.20% | -0.03% | 0.28% | -0.76% |
| Methadone ER Generics | 0.20% | 0.29% | 0.91% | 0.31% | 0.34% | 0.32% | 0.80% | 0.38% |
| Morphine ER Generics | 3.81% | 1.26% | -0.51% | 0.50% | 0.78% | 0.43% | 5.12% | 0.38% |
| Oxymorphone ER Generics | 0.56% | 0.48% | 0.03% | 0.11% | -0.03% | 0.60% | 0.67% | 0.16% |

ER: extended release; ER opioid: extended-release opioid; PA: prior authorization; ST: step therapy; NF: non-formulary
*% change in market share is from pre 6 months to post 6 months following the OER restrictions

In the Regional Commercial PA, OER market share was almost unchanged (dropped by 0.3% from 39.7% to 39.4%) during the 6-month post-restriction period. During the same period, the control plan had a 0.2% increase in OER market share. In this Regional Commercial PA plan restricting OER, market share for other ER opioids also remained relatively constant during the same time period.

Similar to the Regional Commercial PA, there was very little OER share change in National Commercial ST and the control plan during the 6-month post restriction period--OER market share dropped by only 0.3% (from 22.7% to 22.4%) and 0.6% in the case and control plans, respectively. In this National Commercial ST plan, the market share was shifted marginally towards multiple ER opioids, with increases ranging from 0.01% to 0.8%.

The National Medicare NF plan showed the greatest market share change, with the OER share dropping by 9.2% (from 23.5% to 14.3%) during the 6-month post-restriction period; during the same period, the control plan had only a 0.02% decrease in OER market share. In this National Medicare NF plan, the market share shifted in large part towards morphine ER generics, with a 5.1% increase.

**Healthcare Resource Utilization Changes**

Difference-in-differences analyses indicated that overall, there were small and non-significant decreases in PPPM office visits in National/Regional Commercial PA and National Medicare NF plans (-0.1 and -0.2, P>0.05), while a significant, small increase in PPPM office visits in the National Commercial ST plan (+0.1, P=0.0001). Detailed resource utilization changes for each study plan are described below.

In the National Commercial PA plan, PPPM office visits decreased by 0.2 (P=0.012) from the 6-month pre-restriction period to the 6-month post-restriction period, whereas the control cohort decreased by 0.1 visits (P=0.0534) (Table 4). This resulted in a non-significant 0.1 net decrease in PPPM office visits in the case plan after taking into account the decrease in the control plan (P=0.2499). Analysis of the National Commercial PA plan also found that PPPM utilization of all prescription use decreased by 0.2 prescriptions compared to a 0.4 decrease among controls (P<0.0001). This resulted in a 0.2 (P=0.0251) increase in PPPM prescriptions in the case plan after taking the decrease in control use into account.

In Regional Commercial PA plan, the PPPM office visits increased by 0.1 (P=0.0216) from the 6-month pre-restriction period to the 6-month post-restriction period, while the control cohort increased by 0.2 visits (P=0.0002) in the same time period. The net decrease in PPPM office visits for the case plan (after taking into account the decrease in controls) was 0.1, and was not significant (P=0.0709). The case plan PPPM for all prescription use decreased by 0.2 prescriptions (P<0.0001) compared to a 0.1 prescription decrease among controls (P=0.0991). This resulted in a net decrease in PPPM prescriptions of 0.1 (P=0.0444) for the case plan after taking into account the decrease in control plan prescriptions.

In the National Commercial ST plan, PPPM office visits increased by 0.03 (P=0.0624) from the 6-month pre-restriction period to the 6-month post-restriction period, while decreased by 0.1 visit (P=0.0002) in the control plan. After taking into account the decrease in controls, the net increase in PPPM office visits for the case plan was 0.1 and was significant (P=0.0001). The case plan PPPM for all prescription use increased by 0.04 prescriptions (P=0.0004), compared to a 0.3 prescription increase in the control plan (P<0.0001). This amounted to a -0.26 difference between changes in the case vs the control plan (P<0.0001).

The National Medicare NF plan had a decrease of 0.1 PPPM office visits (P=0.1678) from the 6-month pre-restriction period to the 6-month post-restriction period, whereas the control cohort increased by 0.04 visits (P=0.5685) in the same period. This amounted to a non-significant 0.2 (P=0.1604) net decrease in PPPM office visits for the case plan after taking into account the decrease in controls. In this case plan, the PPPM for all prescription use decreased by 0.1 prescriptions (P=0.1837) compared to a 0.2 prescription decrease in the control plan (P<0.0001), resulting in a 0.20 difference between changes in the case vs control plan (P<0.0001).

**Table 4.** Resource Utilization Changes Across Health Plans Before and After OER Formulary Restrictions

| | National Commercial PA | | National Commercial Controls | | | | Regional Commercial PA | | Regional Commercial Controls | | | |
|---|---|---|---|---|---|---|---|---|---|---|---|---|
| | Δ Mean (post-6m – pre-6m) | P-value | Δ Mean (post-6m– pre-6m) | P-value | Δ Mean difference (cases-controls) | P-value | Δ Mean (post-6m– pre-6m) | P-value | Δ Mean (post-6m– pre-6m) | P-value | Δ Mean | P-value |
| **Resource Utilization PPPM** | | | | | | | | | | | | |
| Office Visits | -0.21 | 0.0115 | -0.09 | 0.0534 | -0.12 | 0.2499 | 0.09 | 0.0216 | 0.21 | 0.0002 | -0.12 | 0.0709 |
| All Rx | -0.20 | <0.0001 | -0.35 | <0.0001 | 0.15 | 0.0251 | -0.20 | <0.0001 | -0.08 | 0.0991 | -0.13 | 0.0444 |
| ER opioid Rx | -0.09 | <0.0001 | -0.09 | <0.0001 | 0.01 | 0.6485 | -0.07 | <0.0001 | -0.08 | <0.0001 | 0.01 | 0.3100 |
| Non-opioid Rx | -0.02 | 0.5861 | -0.14 | 0.0008 | 0.12 | 0.0346 | -0.05 | 0.1365 | 0.07 | 0.1093 | -0.12 | 0.0287 |
| SAO Rx | -0.09 | <0.0001 | -0.11 | <0.0001 | 0.02 | 0.0905 | -0.08 | <0.0001 | -0.06 | <0.0001 | -0.02 | 0.2247 |
| **Costs PPPM** | | | | | | | | | | | | |
| Total Costs | -273.01 | NA | -38.18 | NA | -234.83 | NA | -16.18 | 0.7629 | 43.69 | 0.4151 | -59.87 | 0.4296 |
| Office Visits | -$274.04 | 0.0008 | -$46.77 | 0.4417 | -$227.27 | 0.0281 | -2.72 | 0.9587 | 48.62 | 0.3478 | -51.33 | 0.4862 |
| All Rx | $1.03 | 0.932 | $8.59 | 0.6018 | -$7.56 | 0.7031 | -13.46 | 0.2650 | -4.92 | 0.7198 | -8.54 | 0.6402 |
| ER opioid Rx | -$22.24 | <0.0001 | -$15.79 | 0.0007 | -$6.45 | 0.3622 | -5.92 | 0.1121 | -12.42 | 0.0009 | 6.51 | 0.2179 |
| Non-opioid Rx | $4.12 | 0.6642 | $5.81 | 0.6974 | -$1.69 | 0.9158 | -7.96 | 0.4803 | 12.12 | 0.3485 | -20.08 | 0.2416 |
| SAO Rx | $19.16 | <0.0001 | $18.57 | <0.0001 | $0.59 | 0.3003 | 0.41 | 0.6889 | -4.62 | 0.0075 | 5.03 | 0.0122 |
| | **National Commercial ST** | | **National Commercial Controls** | | | | **Regional Commercial PA** | | **Regional Commercial Controls** | | | |
| | **Δ Mean (post-6m – pre-6m)** | **P-value** | **Δ Mean (post-6m– pre-6m)** | **P-value** | **Δ Mean difference (cases-controls)** | **P-value** | **Δ Mean (post-6m– pre-6m)** | **P-value** | **Δ Mean (post-6m– pre-6m)** | **P-value** | **Δ Mean** | **P-value** |
| **Resource Utilization PPPM** | | | | | | | | | | | | |
| Office Visits | 0.03 | 0.0624 | -0.05 | 0.0002 | 0.07 | 0.0001 | -0.12 | 0.1678 | 0.04 | 0.5685 | -0.16 | 0.1604 |
| All Rx | 0.04 | 0.0004 | 0.30 | <0.0001 | -0.26 | <0.0001 | -0.05 | 0.1837 | -0.24 | <0.0001 | 0.20 | <0.0001 |
| ER opioid Rx | -0.05 | <0.0001 | -0.04 | <0.0001 | -0.01 | <0.0001 | -0.10 | <0.0001 | -0.09 | <0.0001 | -0.01 | 0.3314 |
| Non-opioid Rx | 0.12 | <0.0001 | 0.35 | <0.0001 | -0.23 | <0.0001 | 0.12 | 0.0002 | -0.09 | 0.0027 | 0.21 | <0.0001 |
| SAO Rx | -0.03 | <0.0001 | -0.02 | <0.0001 | -0.01 | 0.0003 | -0.06 | <0.0001 | -0.06 | <0.0001 | 0.00 | 0.8183 |
| **Costs PPPM** | | | | | | | | | | | | |
| Total Costs | 44.53 | 0.0114 | 93.27 | <0.0001 | -48.74 | 0.0584 | -17.06 | 0.669 | -54.95 | 0.1926 | 37.90 | 0.5139 |
| Office Visits | 1.56 | 0.9210 | -4.54 | 0.7894 | 6.09 | 0.7922 | 10.25 | 0.7903 | -62.46 | 0.1308 | 72.71 | 0.1982 |
| All Rx | 42.97 | <0.0001 | 97.80 | <0.0001 | -54.83 | <0.0001 | -27.31 | 0.0028 | 7.51 | 0.3115 | -34.82 | 0.0031 |
| ER opioid Rx | -7.57 | <0.0001 | -3.99 | <0.0001 | -3.58 | 0.0026 | -30.92 | <0.0001 | -9.81 | <0.0001 | -21.11 | 0.0006 |
| Non-opioid Rx | 42.79 | <0.0001 | 102.70 | <0.0001 | -59.92 | <0.0001 | 5.44 | 0.4439 | 14.83 | 0.0172 | -9.39 | 0.3199 |
| SAO Rx | 7.76 | 0.0031 | -0.91 | 0.5322 | 8.66 | 0.0039 | -1.83 | 0.0206 | 2.49 | 0.3472 | -4.32 | 0.1182 |

ER opioid: extended-release; PA: prior authorization; PPPM: per-patient per-month; Rx: prescription; SAO: short-acting opioid
All costs were adjusted to 2014 dollars using the Medical Care Consumer Price Index for All Urban Consumers.

### Cost Changes

Difference-in-differences analysis suggests that no significant monthly total cost changes were observed in the post-restriction period for Regional Commercial PA ($59.87 decrease, P=0.4296), National Commercial ST ($48.74 decrease, P=0.0584), and National Medicare NF ($37.90 increase, P=0.5139) plans (Figure 1). However, a significant net decrease in PPPM office visit cost ($227.27 decrease, P=0.0281) in the post-restriction period was observed in the National Commercial PA plan (Table 4).

From the 6-month pre-restriction period to the 6-month post-restriction period, PPPM costs for office visits among the National Commercial PA plan cohort decreased by $274.04 (from $1441.73 to $1167.69; P=0.0008); among the control plan cohort, PPPM costs decreased by $46.77 (from $1118.87 to $1072.10; P=0.4417). This resulted in a significant net decrease of $227.27 (P=0.0281) in office visit costs for the case plan post-restriction after taking into account the decrease in controls. In this comparison, the PPPM costs for all prescriptions remained approximately the same for both case and control patients.

In the Regional Commercial PA plan, PPPM total costs decreased by $16.18 (from $1247.72 to 1231.54; P=0.7629) from the 6-month pre-restriction period to the 6-month post-restriction period, compared with an increase of $43.69 (from $1416.50 to $1460.20, P=0.4151) in the control plan. This resulted in a non-significant post-restriction net decrease of $59.87 (P=0.4296) in total costs for the case plan (after taking into account the decrease in controls). There was a non-significant net decrease of $51.33 (P=0.4862) in the PPPM office visit cost, while the PPPM costs for all prescriptions remained almost the same for both case and control patients.
PPPM total costs increased by $44.53 (from $2245.66 to $2290.19; P=0.0114) in the National Commercial ST plan from the 6-month pre-restriction period to the 6-month post-restriction period; in the control cohort, the PPPM total costs increased by $93.27 (from $1947.17 to $2040.44; P<0.0001). This resulted in a non-significant net decrease of $48.74 (P=0.0584) in total costs for the case plan post-restriction after taking into account the decrease in controls. There was a non-significant net increase of $6.09 (P=0.7922) in the PPPM office visit cost, while the net PPPM costs for all prescriptions decreased significantly by $54.83 (P<0.0001).

In the National Medicare NF plan, PPPM total costs decreased by $17.06 (from $1992.16 to $1975.10; P=0.6690) from the 6-month pre-restriction period to the 6-month post-restriction period; PPPM total costs decreased by $54.95(from $1829.14 to $1774.18; P=0.1926) in the control plan. There was a non-significant net increase of $37.90 (P=0.5139) in PPPM total costs after taking into account the decrease in controls post-restriction. There was a non-significant net increase of $72.71 (P=0.1982) in the PPPM office visit cost, while the net PPPM costs for all prescriptions decreased significantly by $34.82 (P=0.0031).

## DISCUSSION

Managed care organizations use formulary restrictions to influence prescribing behavior and medication utilization. It is documented in the literature that formulary restriction policies, while potentially effective as short-term cost containment measures may also have unintended consequences such as delayed care, negative impacts on patient health status, as well as increased utilization and total costs for disease-related care.[7,13,14] This study evaluated the impact of PA, ST, and NF formulary restrictions applied to OER. The scenarios of interest were PAs implemented in both national and regional commercial health plans, ST in national commercial plans, and NF restrictions in national Medicare plans. The outcomes of interest measured were market share change and most importantly, impact of healthcare resource utilization and costs.

Formulary restrictions tend to shift utilization to preferred products based on restriction type and drug class.

The results from the current study demonstrated modest decreases in market share and utilization when PA, ST, or NF were applied to OER, although changes in some restriction types were negligible. In the 6-month period following the institution of formulary restrictions, decreases in market share were largest for national Medicare plans with NF (9.2 % decrease) and national commercial plans with PA (7.0% decrease); decreases were minimal for regional commercial plans with PA (0.3%) and national commercial plans with ST (0.3%). It should be noted that the regional commercial plan with PA imposed the restriction on new OER prescription starts while allowing existing users to continue (often referred to as grandfathering), which may have contributed to the minimal market share change observed. These findings may also suggest the differential impact of formulary restriction by restriction type and geography-- PA and NF (compared to ST), and national (compared to regional) were associated with a greater impact in reducing OER utilization.

One principle suggests that formulary restrictions leading to decreased utilization of the targeted drug(s) should lead to overall decreases in related healthcare costs; however, difference-in-differences approach employed in this study showed that the effects on total monthly costs following formulary restrictions were inconsistent across plans, and insignificant for most plans. The largest cost reduction was observed in the national commercial plan with PA that saw a net pre-to-post restriction decrease in PPPM office visit costs of $274.00 ($234.83 after adjusting for changes in the control plan). However, no significant net change in pre-to-post restriction office visit costs were observed in the other three study plans. Results on pharmacy costs were mixed. Overall pharmacy costs remained approximately the same in the post-restriction period for national/regional commercial PA plans, while significant decreases were observed in national commercial ST and national Medicare NF plans.

While studies specifically evaluating formulary restrictions placed on OER and other ER opioids in commercial or Medicare plans are limited, the current findings are consistent with two previous evaluations of the effects of various formulary restrictions on OER. An earlier study by Ben-Joseph et al found mixed results regarding the effects of PA and formulary tier changes (TC) for OER in both commercial (regional, national) and Medicare plans. While OER utilization decreased in some commercial and Medicare plans with PAs or STs, there was increased OER utilization in commercial plans with TC. That study also identified significant increases in outpatient office visits and SAO prescriptions.[9] Similarly, a 2008 study by Morden et al reported mixed effects of PAs on branded oxycodone HCl controlled-release (CR) in 49 state Medicaid plans. The study found that while a few states with PAs achieved a significant decrease in branded oxycodone CR utilization, most did not.[15]

Although current study results include a mix of changes in OER market share, significant and non-significant pre to post-index changes in PPPM cost, and variations in these measures among plan types, in general our findings are supported by prior research examining the effects of formulary restrictions on other types of chronic pain medications. Pregabalin is a non-opioid drug used to treat diabetic neuropathic pain, fibromyalgia, and post-herpetic neuralgia, which are all considered chronic pain conditions. Three studies, by Udall et al and Suehs et al, and Margolis et al evaluated the effects of ST and/or PAs on pregabalin in national commercial plans and Medicare managed care plans.[8,16,17] While all three studies reported significant decreases in pregabalin utilization after formulary restrictions were in place, there were no reductions in total healthcare costs. The Udall study actually reported a significant increase in both all-cause and disease-specific total costs.[16]

Various theories have been postulated for the mixed utilization and cost results observed when formulary restrictions are placed on medications used to manage chronic pain conditions. This may be due in part to the unique complexity of chronic pain management. Individual differences in metabolism, environment, comorbidities, psychological condition, and pain etiology make effective management of chronic pain challenging.[10,18] Additionally, since patients may experience a greater heterogeneity of response and side effects to opioids, trial of multiple agents may be required to find an effective medication regimen.

Morden et al suggests that, compared with other drugs, OER appears to be relatively refractory to Medicaid PA restrictions as patients are particularly invested in maintaining the effectiveness of their treatment, rather than seeking out less expensive alternatives.[15] Huskamp et al noted a similar trend in psychotropic drugs and mental illness; patients often exhibit varying responses to the same psychotropic medications. Thus, the difficulty of finding the right treatment match may make patients and providers less willing to switch medications in spite of formulary restrictions.[4]

While the methods in this study do not duplicate previously published work on the impact of formulary restrictions, this study also suggests that there may be unintended economic consequences associated with formulary restrictions for OER due to patients switching to other ER opioids such as morphine ER. Changing opioid regimens is a complex task that may require frequent dose titration, adjustments and careful monitoring, which could lead to additional office visits and attendant costs.[15] In addition, each change in these opioid prescriptions requires a new written prescription. Patient burden associated with formulary changes should also be considered in the implementation of formulary restrictions. The time spent by patients and providers resolving barriers to drug access may lead to delays in medication use, additional costs, reduced adherence and poor patient satisfaction.[19]

**Study Strengths and Limitations**

Some data were unavailable through the claims databases used in the study; this included out-of-network, unrecorded observations, and inpatient hospital encounters, all of which could lead to cost underestimation. Likewise, the 6-month post-restriction observation timeframe may be inadequate to fully capture the potential long-term effects on utilization and cost of care following the institution of formulary restrictions. While PA, ST and NF are all types of utilization management, they are distinctive types of formulary restrictions, and direct comparisons between them are not appropriate. As the formulary restrictions were not all implemented at the same time across the plans studied, market factors such as new product introductions may have influenced market share changes. In addition, due to data availability, it was not possible to control for the full OER formulary history and plan size between cases and controls , which may have played a factor in post restriction patient behaviour. Other factors not considered in this study that may have affected the true cost impact of formulary restrictions are formulary status and rebates available for other ER opioids, health plan call center volume increases, member disenrollment, provider/patient disruption, the cost of formulary change notifications, and other administrative costs.

It has been suggested that modest OER market share reductions and savings in the national and regional commercial plans with PA may be offset by the administrative cost of implementing the PA, as well as the potential loss of rebates, thus resulting in net cost-neutrality.[20] While the effects of administrative costs and rebate loss were not evaluated in this study, prior research has demonstrated that providers spend a substantial amount of time navigating restrictions in varying plans.[19] These costs have been estimated to range from $10 to $75 per event.[21,22]

As this was a retrospective analysis, associations can be observed but causality cannot necessarily be inferred. Therefore, these study results should be considered preliminary and further research with longer follow-up periods, different populations, and other formulary restriction types may be warranted.

## CONCLUSION

While very few studies have evaluated the effect of various types of OER formulary restrictions on market share, healthcare resource utilization and costs across a variety of health plans, the results of this study suggest that formulary restrictions such as PA, ST and NF, while altering OER market share to some extent, may not result in substantial pharmacy/medical cost savings. This study provides further evidence that the impact of formulary restrictions may differ by restriction type and between health plan types and delivers real-world insight for policy and healthcare decision-makers regarding the impact of formulary restrictions on ER opioid utilization and associated costs.

## ACKNOWLEDGMENTS

The authors would like to thank Kainan Sun who provided programming and advanced analytical assistance throughout the course of the study.

## DISCLOSURES

APD, C-CC and RW are employees of QuintilesIMS, a for-profit company, which was contracted by Purdue Pharma to undertake this research. JCH is an employee of Purdue Pharma L.P.
